# Letter from Tama City, Iowa

**Published:** 1882-11

**Authors:** F. W. Gooding

**Affiliations:** Tama City, Iowa


### ARTICLE XIII.

TAMA CITY, IOWA, Oct. 16, 1882.

EDITORS JOURNAL AND EXAMINER:

From the language used by Dr. Theodore Turnbull, of Monticello, Florida, in the August number of the JOURNAL, I judge that he believes quinine will, and is very liable to, produce abortion. I have given quinine to five pregnant females, in doses ranging from 15 grains to 40 grains per diem in divided quantities, in intermittent and remittent fevers, without the first untoward symptom, each case terminating in a recovery, while gestation continued to full term. One of the cases—a member of my own family—was troubled with "chills and fever," suffering quite severely from the fifth to the seventh month of pregnancy without obtaining the least benefit from a faithful trial of the different substitutes for the cinchona alkaloid. I had read that quinine was "a stimulant to the muscular fibers of the uterus," hence I concluded it would be dangerous to use it under the above circumstances. I remembered my worthy professor of materia medica and therapeutics, as saying, that "quinine, although a stimulant to the uterine muscular fiber, was rarely certain in its influence except when that organ was in a gravid state; and then, the nearer the time of confinement the more certain its action." He then recommended it in slight post partum hæmorrhages. After thinking it carefully over, I concluded to risk it, as the patient was becoming quite ænemic and did so. The malarial influence was rapidly driven out of the system and now—eighth month of her pregnancy—she is enjoying excellent health with no return of the trouble, under the pernicious influence of from 40 grains diminished to 10 grains per diem.

I have asked several of the oldest physicians in this (Tama) county, and all say they use quinine without reference to the pregnant or non-pregnant state.

F. W. GOODING, M.D.

